# Assessing spatio-temporal patterns and driving force of ecosystem service value in the main urban area of Guangzhou

**DOI:** 10.1038/s41598-021-82497-6

**Published:** 2021-02-04

**Authors:** Yi He, Wenhui Wang, Youdong Chen, Haowen Yan

**Affiliations:** 1 Faculty of Geomatics, Lanzhou Jiaotong University, Lanzhou, 730700 China; 2 National-Local Joint Engineering Research Center of Technologies and Applications for National Geographic State Monitoring, Lanzhou, 730700 China; 3 Gansu Provincial Engineering Laboratory for National Geographic State Monitoring, Lanzhou, 730700 China

**Keywords:** Ecosystem services, Urban ecology, Ecology

## Abstract

Increasing human activity around the world has greatly changed the natural ecosystem and the services it provides. In the past few decades, a series of significant changes have taken place in land use/land cover (LULC) in China due to the rapid growth in population, particularly in the cities of the Zhujiang Deita. However, there have been few attempts to study the co-evolution of land use/land cover change and ecosystem service value (ESV) in the main urban area of Guangzhou. Therefore, based on Landsat TM/OLI images from 1987, 1993, 1999, 2005, 2011 and 2017, the weight vector AdaBoost (WV AdaBoost) multi-classification algorithm was utilized to extract LULC data sets, and the spatiotemporal patterns of LULC over these periods were studied. The ESV was estimated and the driving force was analysed. The effect of LULC dynamics on the ESV was evaluated. The results showed that great changes have taken place in LULC in the main urban area of Guangzhou from 1987 to 2017, of which the most significant was the large-scale expansion of the built-up area that occurred through degradation of the forest and cultivated land. The proportion of forest and cultivated land decreased from 43.12% and 34.23% to 25.88% and 12.59%, respectively. The results between periods revealed a decrease in total ESVs from 5.63 × 10^9^ yuan in 1987 to 5.27, 4.16, 4.62, 3.76 and 4.47 × 10^9^ yuan in 1993, 1999, 2005, 2011 and 2017, respectively. In total, ESVs decreased by 1.16 billion yuan (20.61%) from 1987 to 2017. Water supply, food production, nutrient cycling and gas regulation were the four principal ecosystem service functions that affected the total ESVs. Forest, water body and cultivated land areas played a key role in ecosystem services. Therefore, we advocate that when protecting natural ecosystems in the future land use management in Guangzhou should be prioritized.

## Introduction

Ecosystem services refer to the continuous provision of ecosystem goods and services by ecosystems and their ecological processes. These services comprise and maintain the environmental conditions and material basis upon which human beings depend for survival^1,2^. There are four types of ecosystem services—provisioning services, regulating services, cultural services and supporting services. These are necessary for maintaining other services^3,4^. However, with the constant growth in the global population, industrialization and urbanization, the ecosystem is constantly being encroached upon^5,6^. With the increasing scarcity of ecosystem services, how to protect the ecosystem and improve the supply of ecosystem services has become a common challenge facing the whole world^2^. Therefore, many scholars have devoted themselves to quantifying the value of ecosystem services and evaluating and estimating these services on a global scale^4,7,8^. Based on the evaluation model proposed by Costanza, Xie et al.^4^ proposed an equivalent scale of ecosystem service value (ESV) per unit area of different terrestrial ecosystems in China. This has been widely used in China because of its simplicity and reliability^9^.

Land use/land cover change (LUCC) is a main driving factor in global environmental change^10–12^and ecosystem services change^9^. LUCC leads to changes in ecosystem services by changing the structure and function of the ecosystem^13^. Therefore, as a direct driver of changes in ecosystem service functions, LUCC plays an important role in maintaining and regulating ESV^14^. Land use/land cover (LULC) has changed significantly due to population growth, economic development and urban expansion. Globally, the transformation of natural ecosystems into farmland and urban areas has resulted in an increase in food production, timber, housing and other commodities. These changes have in turn affected numerous ecosystem services^15^. However, the main challenge is that many of the services offered by these ecosystems are public goods not captured by the market. Therefore, they have no monetary value^7,16^^.^ Thus, quantifying LUCC and ESV and analysing their relationship is an essential decision support tool for sustainable use and development.

Cities are the most intensive areas of human activity, and the natural ecosystem is significantly transformed by such activity. Cities also have the most significant impact on the ecosystem service function. Therefore, there is a close relationship between urban land use change and the ecosystem service function^17^. China is undergoing unprecedented urbanization, with the urbanization rate rising from 17.92% in 1978 to 58.52% in 2017^18^. This rapid urbanization process will continue to evolve in the coming decades, and it is expected that by 2035–2040 urbanization in China will reach 70%^19^. In the process of promoting the construction of ecological civilization, such as capitalization management of natural resources and ecological compensation, it is imperative to evaluate ESV. The Zhujiang Delta is the region with the most developed economy, the densest population and the fastest urbanization. At present, the level of urbanization has reached about 70%, which is obviously higher than the national average^16,20^. This rapid urbanization not only promotes rapid social and economic development but also weakens the vital ecological services provided by the natural ecosystem for the city^21^. As one of the rapidly urbanized areas in the Zhujiang Delta, the main urban area of Guangzhou is facing increasing pressure on resources and the environment due to the rapid development of the social economy in recent years.

Guangzhou has seen massive changes in LULC in recent years^22,23^. For example, Li and Cui^22^ analyzed spatiotemporal patterns based on thematic mapper (TM) images from 1990 to 2006 and found that the growth of construction land was most significant, as a large amount of cultivated land, forest, grassland and water-covered areas were converted into construction land. He et al.^23^ studied LUCC in the main urban area of Guangzhou based on Landsat images. Since 1987, cultivated land has disappeared, vegetation has decreased and urban land areas have doubled. In addition, there were some studies on LUCC and ESV in Guangzhou, Ye et al.^24^ quantified land-use change and valued the impact on ecosystem services from 1990 to 2010 in the rapidly urbanizing Guangzhou-Foshan Metropolitan Area, southern China. Gao et al.^25^ studyied urban land-use planning under multi-uncertainty and multiobjective considering ecosystem service value and economic benefit in Guangzhou, China. Hu et al.^26^ studied spatio-temporal changes in ecosystem service value in response to land-use/cover changes in the Pearl River Delta from 1995 to 2015. Ye et al.^27^ analyzed the landscape pattern changes, the dynamics of the ESVs and the spatial distribution of ESVs from 1995 to 2005 in Guangzhou, which is the capital of Guangdong Province and a regional central city in South China. It is well known that the progress of urbanization will lead to the change of land use types, this transformation will bring about changes of ESV, which will affect the urban ecological structure and indirectly regulate the types of ecological services^13,15^. It will eventually lead to the deterioration of the ecological environment in the urban area. Although some studies have been conducted on LULC change in this region, it did not further reveal the positive and negative effects of LUCC from the perspective of ESV. In addition, few attempts addressed the dynamics of LULC and ESV with the detailed characteristics in the main urban area of Guangzhou from a long time scale and a short time interval. Further, a quantitative assessment to reveal the impact of LULC changes on ESV is seldom attempted. Thus, we aim at closing the gaps of the previous LULC change studies in the main urban area of Guangzhou by estimating changes in the economic value of ecosystem services. A quantitative analysis is urgently needed in the main urban area of Guangzhou, where the increasing population pressure and the corresponding increasing impact of human activities on the land have resulted in dramatic changes in LULC.

In 2017, the population of Guangzhou’s main urban area was estimated to have increased by 5.31 million^28^. Unprecedented population growth will cause further pressure on limited land resources and the environment. Therefore, it is crucial to systematically monitor the response relationship between LUCC and ESV in the past 30 years. This paper addresses two major questions: (1) What are the dynamic spatiotemporal patterns of LULC in the main urban area of Guangzhou from 1987 to 2017 (2) How does the spatiotemporal patterns of the ESV change and respond to the LULC? To calculate ESV in the main urban area of Guangzhou in the past 30 years, a quantitative evaluation is helpful to provide a scientific basis for the efficient allocation of land resources, rational regional planning and sustainable development, the scientific management of the ecosystem and the scientific formulation of ecological compensation policy.

## Materials and methods

**Figure 1:**
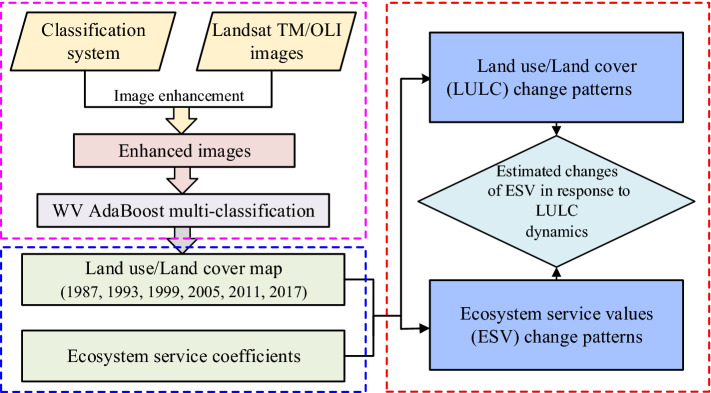
The framework of this paper.

This paper uses Landsat TM/OLI images from 1987, 1993, 1999, 2005, 2011 and 2017 by the weight vector AdaBoost (WV AdaBoost) multi-classification algorithm extracting LULC data sets, and the spatiotemporal patterns of LULC over these periods were analysed. The spatiotemporal change patterns and driving force of ESV was estimated. The effect of LULC dynamics on the ESV was evaluated. The flow chart is shown in Fig. 1.

### Study area

**Figure 2:**
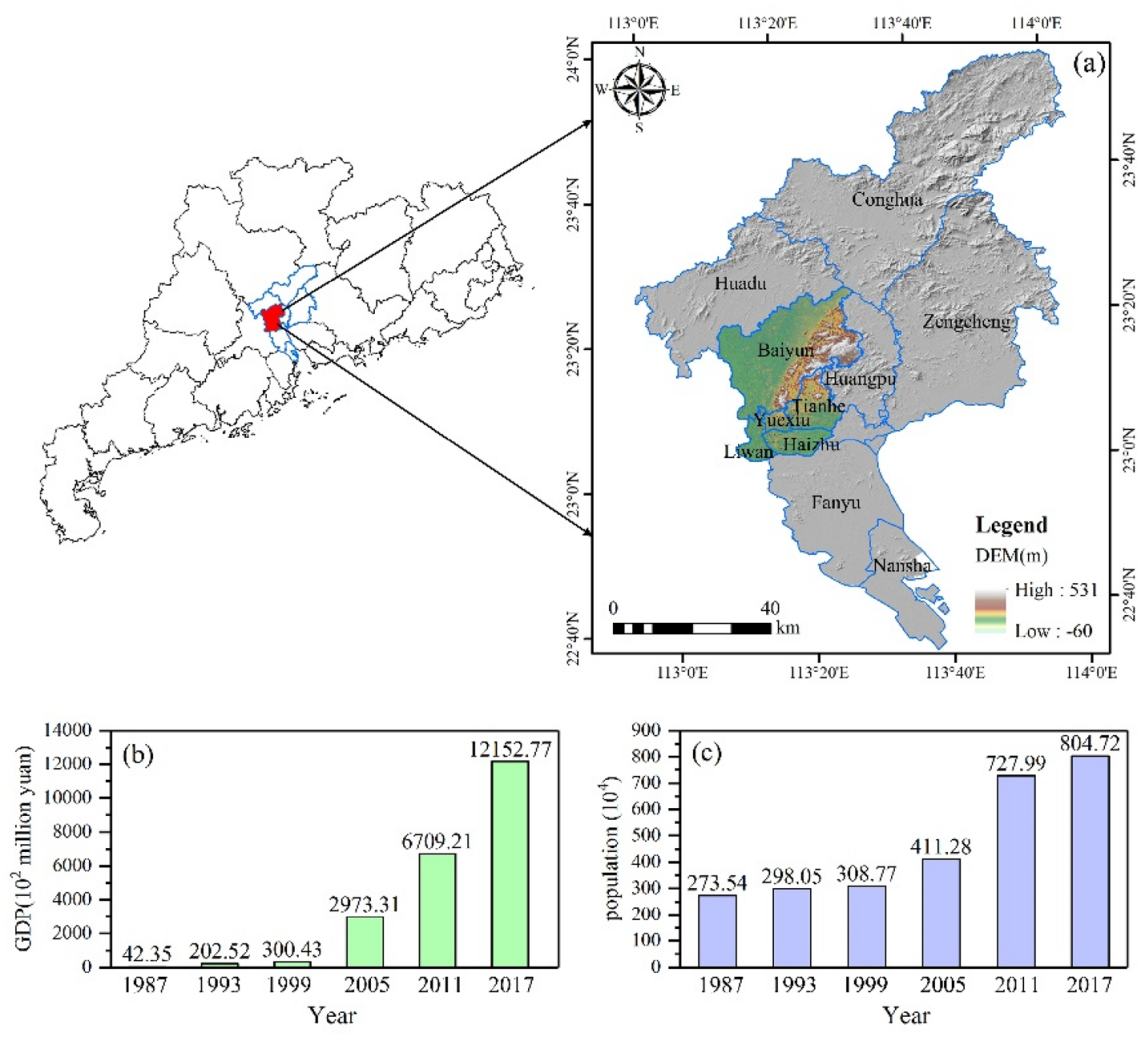
Location of study area and GDP and population. (**a**) location, (**b**) GDP, (**c**) population. (Software: Arc Map 10.5.0, http://www.esri.com. OriginPro 2017C SR2, https://www.originlab.com/).

Guangzhou is located at 112° 57′ ~ 114° 3′ E, 22° 26′ ~ 23° 56′ N in the southeast part of Guangdong Province in the northern margin of the Pearl River Delta. It has a total land area of 7434.40 km^2^. The topography is high in the northeast and low in the southwest, with low mountains and hills in the north and plains in the south and a rich geomorphology. The level of urbanization is high, the land use structure is complex and the land use pattern is changing rapidly. Regional public infrastructure, commercial service land and external transportation land is increasing. Export-oriented industrial agglomeration areas are developing continuously, and the characteristics of export-oriented land use are obvious. Guangzhou has a subtropical oceanic monsoon climate, with an annual average temperature of 20–22 °C and an annual rainfall of about 1720 mm. Guangzhou includes eleven districts (Fig. 2a). This paper focuses on the main urban area of Guangzhou as the research object, a decision based on the city government’s overall urban development strategy for Guangzhou (2010–2020), the main urban area of Guangzhou includes five districts—Liwan, Yuexiu, Haizhu, Baiyun and Tianhe (Fig. 2a). In 2017, the per capita GDP will exceed 136,100 yuan for the first time (Fig. 2b). The population density is large, and the intensity of development is extraordinary. The population has increased from 2.73 million in 1987 to 8.05 million in 2017 (Fig. 2c). The industrial structure has been constantly optimized and improved. The proportion of secondary industries decreased from 31.46% in 1987 to 11.28% in 2017, while the proportion of tertiary industries increased from 61.9% in 1987 to 89.61% in 2017.

### Data and data processing

#### Data

The data used include Landsat series images, an administrative zoning map of Guangzhou, a local historical land use map and social and economic statistics from 1987 to 2017. To explore the socio-economic and natural factors driving the ESV change for different land use types, we examined ten factors—the normalized vegetation index (NDVI), year-end population, elevation, slope, distance from roads, distance from railways, land cover types, GDP, secondary industry GDP and investment in fixed assets.

The Landsat images were taken from the United States Geological Survey website (http://glovis.usgs.gov/), and we downloaded cloud-free Landsat 5 TM and Landsat 8 OLI images (path 122, row 44). Images taken in the dry season (October, November and December) were selected because there was less cloud cover, the change in surface reflectance was much smaller than in other seasons and the image quality was higher. The spatial resolution of imaging is 30 m, and the data of Landsat 5 TM (8 December 1987, 24 December 1993, 25 December 1999, 23 November 2005, 19 November 2011) and Landsat 8 OLI (23 October 2017) were collected.

The elevation data are provided by the Japan Aerospace Exploration Agency (http://www.eorc.jaxa.jp/ALOS/en/aw3d30/data/index.htm); the horizontal resolution is 30 m, and the elevation accuracy is 5 m. The road and railway data are provided by Openstreetmap (download.geofabrik.de/), and the distance between roads and railways were calculated. The land use types are provided by Tsinghua University (http://data.ess.tsinghua.edu.cn/) with a resolution of 10 m. GDP, secondary industry GDP and investment in fixed assets were provided by Guangzhou Statistics Bureau^28^.

Considering the size of the study area and the resolution of the data, we used Fishnet in ArcGIS10.5 to establish a 500 × 500 m grid covering the study area. The values of ten factors were extracted to the grid centre points, and 3915 points were obtained.

#### Remote sensing extraction of LULC types

To obtain high-precision LULC data, the surface features based on the original remote sensing image were enhanced using the index model (the Index-based Built-up Index (IBI)^29^, the modified normalized difference water index (MNDWI)^30^ and the soil-adjusted vegetation index (SAVI)). The humidity, brightness and green chroma indices were transformed using the tasselled-cap method^23^^.^ Using the six indices, six images were calculated and superposed into a new multi-band image in the order of IBI, SAVI, MNDWI, brightness, green chroma and humidity (referred to as the six-index image). This was superposed with the original image (band 1, 2, 3, 4, 5, 7) to create an enhanced 12-band image as the data source for LULC classification. The image stretching function was then used to stretch the composite image to better distinguish the characteristics of land use types. After feature enhancement, the LULC types in the main urban area of Guangzhou could be divided into seven types—forest, water body, grassland, cultivated land, high reflectivity building, low reflectivity building and bare land. Of these, high reflectance buildings and low reflectance buildings are sub-categories of the built-up area category. The spectrum of high reflectivity buildings is similar to that of bare land; therefore, they are classified separately to avoid erroneous classification^31^. Based on Google Earth and TM/OLI images, the interpretation marks of LULC types were established (Table 1). To obtain pure and representative samples, the K-means method of unsupervised classification was utilized to cluster the pixels of the ISM image into seven unlabelled classes and to eliminate abnormal clustering. We then selected uniform points and drew polygons on them. Each polygon contains more than 100 pixels, and each class contains between five and seven polygons. All polygons were saved in a shape file as a mask file, which is utilized to extract pure pixels as training samples.

**Table 1:** Interpretation mark of land cover types.

Land cover types	Description	Thumbnail
Forest	Single green and dark green, fine texture	
Grassland	Green or light green, smooth texture	
Cultivated land	Green or greyish green, with Textured geometry and clear borders	
Built-up area	Subclass high reflectivity buildings, mainly white, distributed and scattered	
Built-up area	Subclass low reflectivity building, mainly grey, rough texture	
Water body	Dark blue, blue and light blue are often shown as curved bands of different widths	
Bare land	Purple or pink, rough texture, multi-block distribution	

Dou and Chen^31^ suggest using the weight vector AdaBoost (WV AdaBoost) multi-classification algorithm for LULC classification. Compared with AdaBoost, it provides higher classification accuracy and stability. WV AdaBoost includes a C4.5 decision tree, a Naïve Bayes neural network and an artificial neural network. In this study, we use Naïve Bayes-based WV AdaBoost to classify LULC based on Landsat-enhanced images. To obtain a highly accurate LULC classification, it is necessary to post-process the image after classification, overlay the early land use map during the research period, check the incorrectly classified area, and filter and correct some segments. After image classification, the random selection of 200 pixels in each class was checked, and the correctness of the classification and the evaluated accuracy were confirmed.

The average classification accuracy of WV AdaBoost based on Naïve Bayes on the original image is 85.02%, and the kappa coefficient is 0.839. The WV AdaBoost algorithm is based on Naïve Bayes processes for the 12-band images of the new combination (enhanced 12-band image). The average classification accuracy is 88.86%, and the kappa coefficient is 0.871. The classification results still need to be strengthened by post-processing, which achieves good classification accuracy. The final average classification accuracy is 91.97%. The kappa coefficient is 0.907. These classification results agree with the ensuing analysis of LULC.

### Methods

Based on Landsat TM/OLI data from 1987 to 2017, a transfer matrix, a land use change index, an ESV evaluation index, a sensitivity model (CS) and a geo-detector (P) were used to analyze the response of ESVs to LULC evolution.

#### Transfer matrix

**Figure 3:**
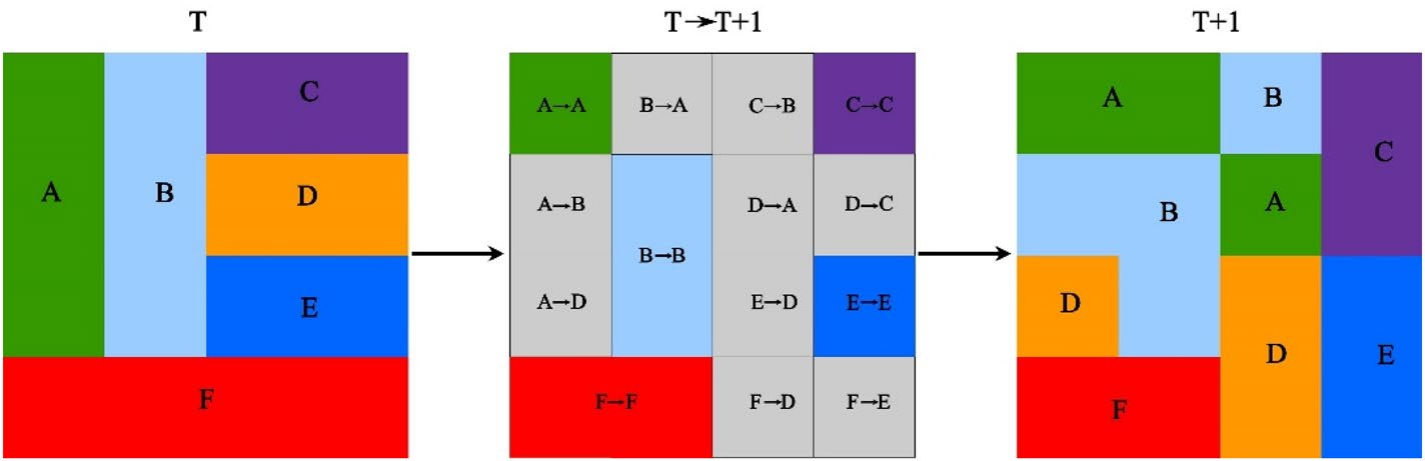
Land use transfer process. *T* the beginning of land use types, *T + 1* the end of land use types; A, B, C, D, E, F: different land use types.

The transfer matrix reflects the dynamic process information about mutual transformation LULC types at the beginning (T) and the end (T + 1) of a specified period of time in a certain region (Fig. 3). The general form is:

1 $${S}_{ij}=\left[\begin{array}{c}{s}_{11} {s}_{12}\dots {s}_{1n}\\ {s}_{21} {s}_{22}\dots {s}_{2n}\\ \dots \dots \dots \dots \\ { s}_{n1} {s}_{n2}\dots {s}_{nn}\end{array}\right]$$

where S stands for area, and i,j (i,j = 1,2,…, n) represents LULC types before and after the transfer.

#### The LULC analyzing indices

1. Land use dynamics (RS)
  Land use dynamics describe the rate and magnitude of LUCC. The general form is:
  2 $$RS=\frac{{U}_{b}-{U}_{a}}{{U}_{a}}\times \frac{1}{T}\times 100\mathrm{\%},$$
  where ${U}_{a}$ is the area of a certain land class at the beginning (km^2^), ${U}_{b}$ is the area of a certain land class at the end (km^2^), and T is the study period.
2. Spatial dynamics of land use (RSS)
  Spatial dynamics of land use describe the degree of spatial change in a certain land use type. The general form is:
  3 $$RSS=\frac{{U}_{in}-{U}_{out}}{{U}_{a}}\times \frac{1}{T}\times 100\mathrm{\%},$$
  where ${U}_{in}$ is the sum area of other types converted to this type in study period *T*, and ${U}_{out}$ is the sum area of a certain type converted to other types in study period *T*. ${U}_{a}$ is the area of a certain type at the beginning.
3. Land use change state index (PS)

The land use change state index represents the trend and state of LUCC. The general form is:

4 $$PS=\frac{{U}_{in}-{U}_{out}}{{U}_{in}+{U}_{out}},$$

where ${U}_{in}$ is the sum area of other types converted to this type in study period *T*, and ${U}_{out}$ is the sum area of a certain type converted to other types in study period *T*.

#### Calculation of ecosystem service value

In this paper, the improved ecosystem services valuation method based on the unit area value equivalent factor proposed by Xie et al.^2^ is employed to evaluate ESV. Therefore, the LULC types of the main urban area of Guangzhou were associated to the corresponding representative biomes (Table 2). The most representative biomes used as a proxy for each LULC type are: (1) cropland for cultivated land, (2) tropical forest for forest, (3) grassland for grassland, and (4) water system and wetland for water body. (5) bare land for bare land.

**Table 2:** Parameters of ESV for different land use types in the main urban area of Guangzhou.

Primary types	ESV calculation index	Forest	Grass land	Cultivated land	Water body	Bare land
Provisioning services	Food production	9.88	3.41	43.94	22.14	0.00
	Raw material	22.48	4.77	4.09	12.26	0.00
	Water supply	11.58	2.73	− 78.01	185.31	0.00
Regulating services	Gas regulation	73.92	17.37	35.77	45.31	0.68
	Climate regulation	221.42	45.65	18.40	100.15	0.00
	Purify environment	65.75	14.99	5.45	155.68	3.41
	Water regulation	161.47	33.38	82.10	2153.93	1.02
Supporting services	Soil conservation	90.27	21.12	4.43	55.19	0.68
	Nutrient cycling	6.81	1.70	6.13	4.09	0.00
	Biodiversity	82.10	19.08	6.81	177.48	0.68
Cultural services	Aesthetic landscape	36.11	8.52	2.73	112.76	0.34

The LULC types are not exactly identical with the representative biomes. For example, cultivated land in this study accounts areas used for paddy fields and dry land. Therefore, the ESV index of cultivated land is calculated by weighting the area ratio of paddy field and dry land in the statistical yearbook. The average value of the ESV index of the water system and wetland is adopted for water body. The ESV index of build-up area is 0. the ESV index of land use types is shown in Table 2. The value of the universal equivalent factor ESV (D value) of the improved ESV method is 340.65 thousand yuan/km^2^.

In this study, the ESV of each land use unit area is based on the research methods of Costanza^3^ and Xie et al.^2^. The calculation formula is:

5 $$ESV=\sum {A}_{i}\times {VC}_{i}$$

6 $${ESV}_{f}=\sum {A}_{i}\times {VC}_{fi},$$

where ESV is the total value (yuan) of ecosystem services, ${A}_{i}$ (km^2^) is the area of class *i*, and *VC*_*i*_ is the ESV coefficient (yuan/km· year) corresponding to class *i*. *ESV*_*f*_ is the single ESV, and VC_fi_ is the value coefficient of the single service function.

#### Sensitivity analysis model

The sensitivity model is used to calculate the response of ESV to the change of value coefficient (VC)^14^ by adjusting the 50% of the ESV coefficient of each land use type up and down and determining the change in ESV over time and the degree of dependence on the value coefficient. The calculation formula is as follows:

7 $$CS=\frac{{(ESV}_{j}-{ESV}_{i})/{ESV}_{i}}{{(VC}_{jk}-{VC}_{ik})/{VC}_{ik}},$$

where ESV is the estimated ESV, VC is the value coefficient, i and j are the initial value and adjusted value (50% up and down adjustment) and K is the land use type. When CS ≥ 1, ESV is elastic relative to VC; when CS ≤ 1, ESV is inelastic. The larger the CS value is, the more critical the accuracy of the ESV index.

#### Grid analysis method

Grid analysis method is used to divided the study area into regular grid matrixes with the same size and no overlap, and takes grid as the research object to express and statistical unit in geospatial space^9^. It uses regular square grid as ESV's spatial statistical unit instead of irregular land-use map spots to ensure the capacity invariance within the unit, which not only highlights the spatial distribution characteristics of ESV, but also facilitates the spatial quantitative statistics of ESV.

The premise of calculating ESV spatial differentiation is to determine the size of grid cell. In this study, based on ArcGIS10.5 software, the area of each land use type in four grid units with side length of 0.5 km, 1 km, 2 km and 3 km was extracted respectively, and then compare the degree of area change, namely the coefficient of variation. The grid of this scale is the optimal grid unit size for the spatial differentiation of ESV in the study area. Finally, the grid analysis method is introduced to construct a (0.5 × 0.5) km square grid as a geospatial statistical unit. By using the equal spacing system sampling method, the study area is divided into 2024 square grids that do not overlap each other (0.5 × 0.5) km, and the grid matrix is composed of these grids. Through the intersection operation of grid matrix and land use data of each research year, the area of various land use types in each grid is counted, and the spatial heterogeneity of land use types and ESV is analyzed.

#### Geo-detector

Combined with GIS spatial superposition technology and set theory, a statistical method proposed by Wang et al.^32^ can detect spatial heterogeneity and reveal the driving force to identify the interaction between multiple factors. This model is widely used to analyze the influence mechanism of social economic factors and natural environmental factors. The geo-detector consists of four detectors—a differentiation and factor detector, an interaction detector, a risk area detector and an ecological detector. In this paper, factor detection and interaction detection are utilized to detect and analyze the driving force of ESVs in the main urban area of Guangzhou.

(1) Factor detector: Factor detection can identify the explanatory power of each spatial driving factor in landscape type change, and its model is as follows:

8 $$P=1-\frac{1}{{n\delta }^{2}}\sum_{i=1}^{m}{n}_{i}{{\delta }^{2}}_{i},$$

where *P* is the explanatory power index of ESV influencing factors; *n*_*i*_ is the number of samples in the secondary area; *n* is the total number of samples; *m* is the number of samples in the secondary area; ${\delta }^{2}$ is the variance in land use type change in the whole area; and ${{\delta }^{2}}_{i}$ is the variance in land use type in the secondary area. Thus, the model is established, assuming ${{\delta }^{2}}_{i}$ ≠ 0.

The range of values for *P* is [0,1]. When *P* = 0, it shows that the spatial distribution of ESV changes is random. The larger the *P* value is, the greater the impact of longitudinal driving factors on ESV changes.

(2) Interactive detector: Interaction detection can be used to identify the interaction between different spatial drivers. When detecting the interaction of X1 and X2, the explanatory power of the dependent variable Y will increase or decrease. The evaluation method is used to calculate the q value of two factors, X1 and X2, for Y, respectively, q (X1) and q (X2); to calculate the q value of their interaction, q (X1 ∩ X2); and to compare q (X1), q (X2) and q (X1 ∩ X2). The five results of the interactive detector are given in Table 3.

**Table 3:** Types of interactions between two covariates.

Criterion	Interaction
q(X1 ∩ X2) < Min(q(X1), q(X2))	Nonlinear weakening
Min(q(X1), q(X2)) < q(X1 ∩ X2) < Max(q(X1)), q(X2))	Single-factor nonlinear weakening
q(X1 ∩ X2) > Max(q(X1), q(X2))	Two-factor enhancement
q (X1 ∩ X2) = q(X1) + q(X2)	Independence from each other
q(X1 ∩ X2) > q(X1) + q(X2)	Nonlinear enhancement

## Results and analysis

### LULC patterns

#### The overall change in LULC

**Figure 4:**
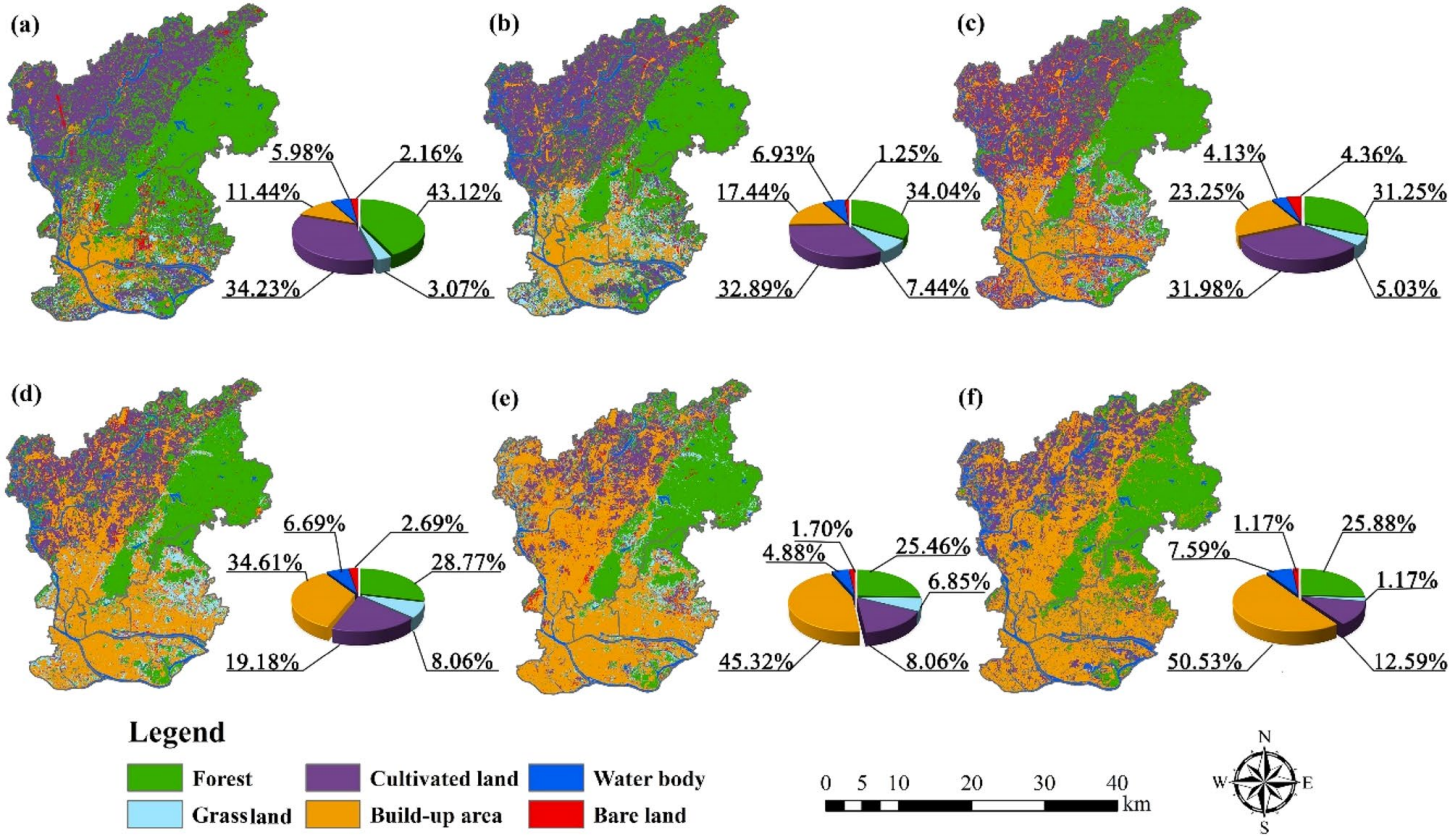
LULC types in the main urban area of Guangzhou from 1987 to 2017. (**a**) 1987, (**b**) 1993, (**c**) 1999, (**d**) 2005, (**e**) 2011, (**f**) 2017. (Software: Arc Map 10.5.0, http://www.esri.com. OriginPro 2017C SR2, https://www.originlab.com/).

From 1987 to 2017, LULC types changed considerably in the main urban areas of Guangzhou, with forest, cultivated land and built-up areas accounting for the major types (Fig. 4). In the past 31 years, the LULC types that changed most dramatically were cultivated land and built-up areas. The cultivated land area declined significantly from 34.23% in 1987 to 12.59% in 2017, while the built-up areas increased remarkably from 11.44% in 1987 to 50.53% in 2017.The proportion of built-up areas is decreasing from the central area to the nearby suburbs and the outer suburbs. Grassland showed the change trend of “increase–decrease–increase–decrease–decrease” between 1987 and 2017, and water body showed the change trend of “increase–decrease–increase–decrease–increase”. Forest showed the trend of gradual decrease. Bare land showed the trend of fluctuation, but the change range was not large and the overall proportion of bare land was small in the study area. In general, the variation in built-up area was the largest and the variation in bare land was the smallest among the LULC types. The continuous decrease in forest and cultivated land and the rapid increase in built-up areas are caused by the rapid economic development of the main urban area of Guangzhou.

#### LULC change between 1987 and 2017

The transfer matrix is utilized to further analyse the change characteristics of each type of LULC in the main urban area of Guangzhou (Table 4). Built-up areas and water body areas increased, while other LULC types decreased between 1987 and 2017. The expanded built-up areas grew rapidly at the rate of 2.41% per year, and the total increased areas equalled 335.90 km^2^, which was 1.59% higher than the water body area growth rate. Grassland decreased by 90.67%, followed by bare land with a decrease of 68.83%, cultivated land with a decrease of 45.26% and forest with a decrease of 37.54%. The proportion of grassland to built-up areas was as high as 77.76%, followed by the proportion of bare land and cultivated land to built-up areas of 65.36% and 51.16%, respectively.

**Table 4:** Conversion matrix of LULC change (km^2^) between 1987 and 2017.

2017	1987						Total area 2017
	FS	GL	CL	BU	WB	BL	
Forest	247.47	1.432	11.445	1.598	1.415	3.271	266.632
Grassland	0.972	0.702	0.746	0.077	0.051	0.286	2.835
Cultivated land	39.193	3.342	130.804	7.080	2.947	2.203	185.568
Build-up area	129.987	23.616	173.437	98.065	10.152	13.977	449.234
Water body	7.177	1.042	19.363	6.167	44.290	1.083	79.122
Bare land	2.096	0.237	3.234	0.344	0.191	0.564	6.666
Total area 1987	426.895	30.371	339.029	113.332	59.046	21.384	990.057
Direction of change	↓	↓	↓	↑	↑	↓	

FS, GL, CL, WB, BU and BL refer to forests, grassland, cultivated land, water bodies, built-up area and bare land, respectively.

**Figure 5:**
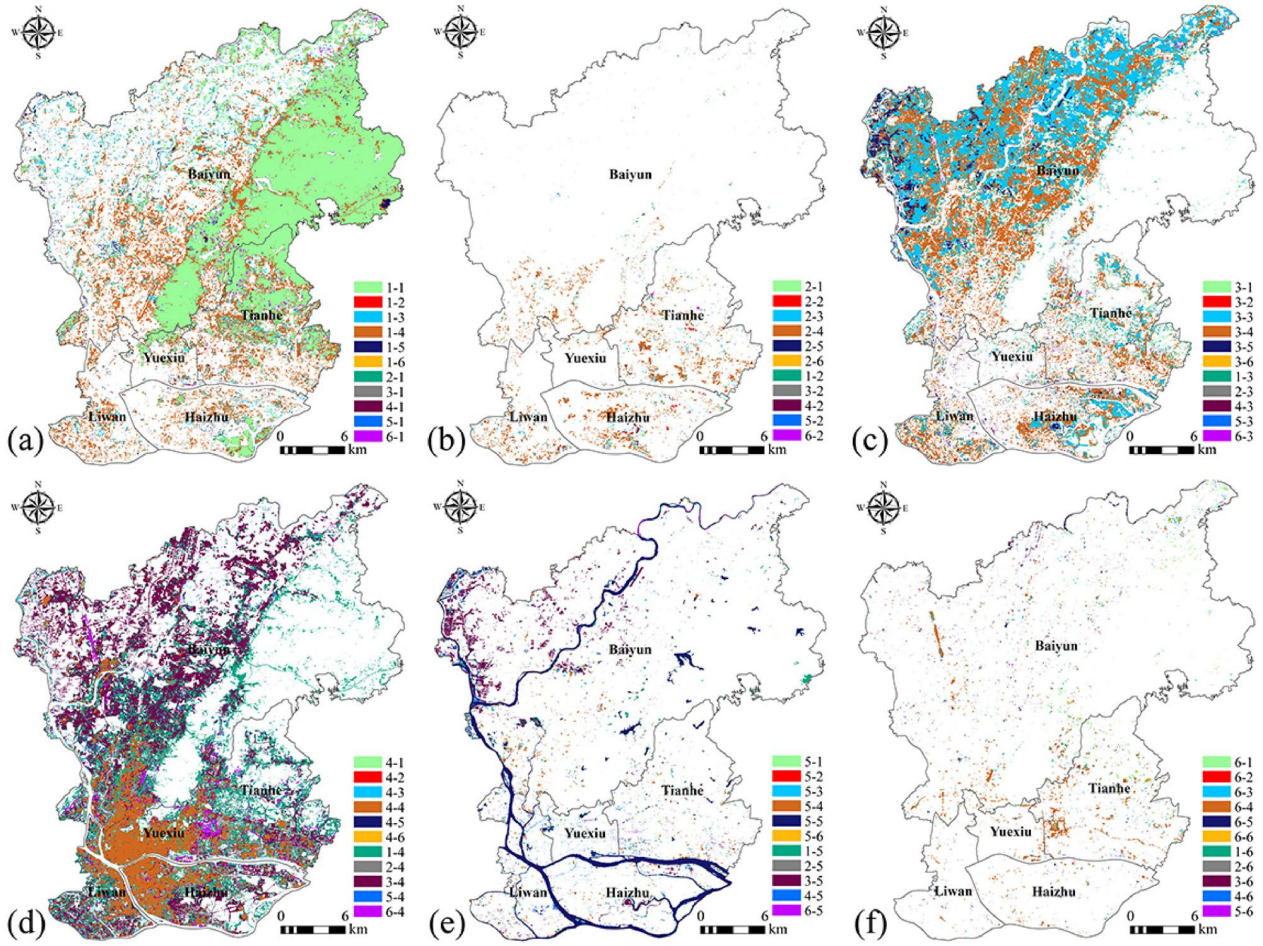
Spatial conversion change of LULC types in the main urban area of Guangzhou between 1987 and 2017. (**a**) forest, (**b**) grassland, (**c**) cultivated land, (**d**) built-up area, (**e**) water body, (**f**) bare land. (Software: Arc Map 10.5.0, http://www.esri.com). 1, 2, 3, 4, 5 and 6 refer to forest, grassland, cultivated land, built-up area, water body and bare land, respectively.

Forest, cultivated land and built-up areas were frequently converted (Fig. 5). The forest transferred 160.26 km^2^ of forest became built-up areas, mainly distributed in the five districts of the main urban area. Most of that forest area was cultivated land mainly distributed in the Baiyun district and had an area of about 39.19 km^2^ (Fig. 5a). A total area of 27.54 km^2^ was transferred from grassland, of which the areas that became build-up area measured 23.62 km^2^ and were distributed in the Tianhe district, the southeast part of the Zhuhai district and the Liwan district (Fig. 5b). Cultivated land is a landscape type with significant changes in the study area. It is mainly located in the northwest part of the Baiyun district and the southeast part of the Haizhu district. The cultivated land was mostly converted into built-up areas, reaching 173.44 km^2^ (Fig. 5c). Built-up areas comprised the most transferred landscape type with a land area increase from 113.33 km^2^ in 2017 to 449.23 km^2^ in 2017. Built-up areas mainly occupied forest land, cultivated land, grassland and other ecological land to achieve space expansion. Change areas were concentrated in the northwest part of the Baiyun district, the Tianhe district, the southeast part of the Zhuhai district and the southern part of the Liwan district, and the landscape space aggregation became increasingly obvious (Fig. 5d). The increase in water body areas was transferred from cultivated land and forest, and the decrease was transferred into built-up areas. The change process was mainly concentrated in the Baiyun district (Fig. 5e). In addition, bare land was primarily converted into built-up areas (Fig. 5f).

#### Dynamic change of LULC

**Figure 6:**
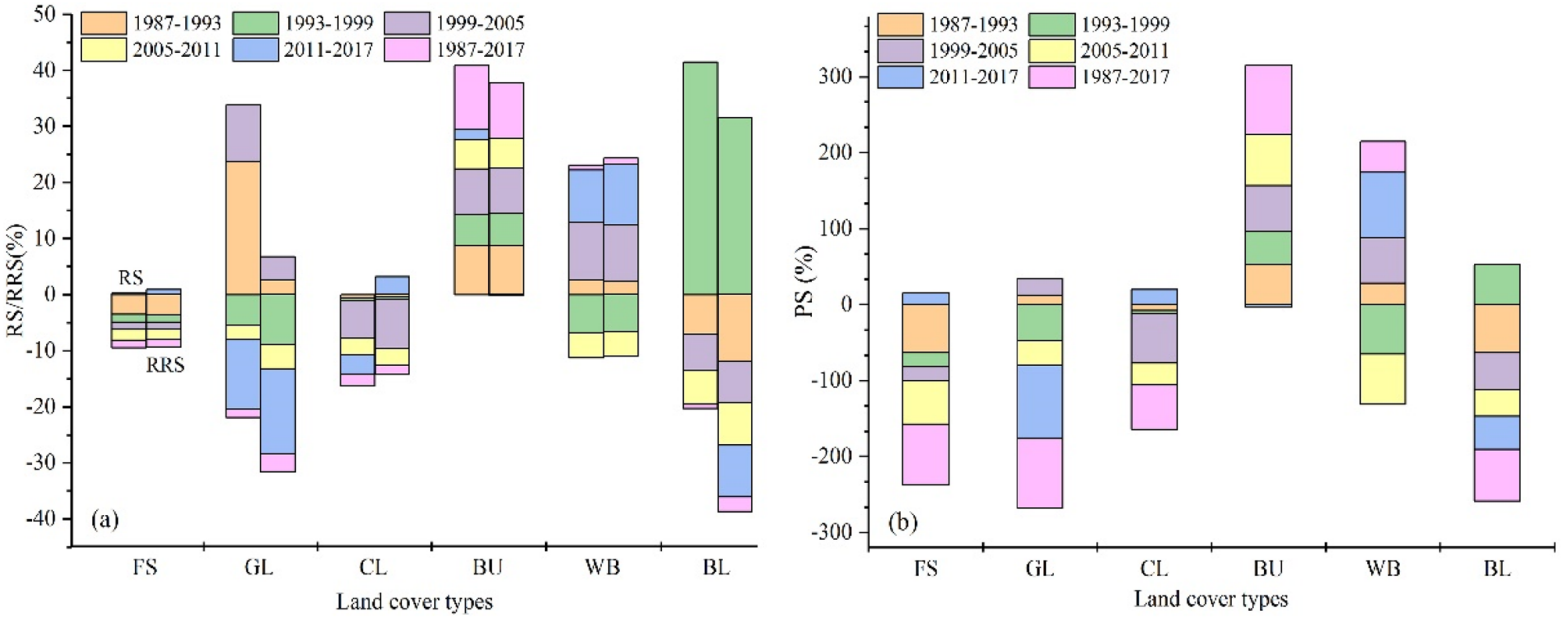
Change of each land cover type in different periods. FS, GL, CL, BU, WB and BL refer to forests, grassland, cultivated land, water bodies, built-up area and bare land, respectively.

According to the LULC dynamic change index from 1987 to 2017 in the study area (Fig. 6), the dynamic degree (RS was 9.88%, RSS was 1.13%) and change status index (PS was 91.67%, 40.49%) of the built-up and water body areas were relatively high in the last 30 years, indicating that they had a large change range, a fast growth rate and a trend of continuous increase in area. During the period from 1999 to 2005, the dynamic degree and change state index of cultivated land were lower than in other periods, while the RSS and PS values of the built-up and water body areas were higher, indicating that the growth rate of cultivated land area was decreasing and the growth rate was slowing down, while the growth rate of built-up and water body areas was accelerating. The RS of forest increased from − 3.51% between 1987 and 1993 to 0.27% between 2011 and 2017, indicating that the growth and increase rates of forest areas were small between 1987 and 1993 but increased between 2011 and 2017 and that the change was drastic in the study time series. In the past 30 years, the spatial dynamic degree of built-up and water body areas was noticeable, indicating that the transfer area of built-up and water body areas was larger and that the space transfer was frequent. However, the dynamic degree of grassland and bare land was less, indicating that the spatial change frequency of these two land use types is trivial. The change in dynamic degree and in the state index for forest areas both showed the trend of “increase–increase–decrease–increase”, indicating that the fluctuation in forest area in the study period had a trend of slowing down. The RSS of cultivated land increased from -0.48% between 1987 and 1993 to 3.18% between 2011 and 2017, indicating that cultivated land conversion was frequent and that the longitudinal change frequency was high between 2011 and 2017 and was stable between 1987 and 1993. The decrease in area of cultivated land was related to the gradual increase in the population’s demand for cultivated land, the low awareness of rational exploitation of resources and the modest awareness of the protection of cultivated land in recent years. From 2011 to 2017, the RSS and PS values of water body areas both increased significantly, indicating the frequent spatial changes in water body areas. This was significantly related to the gradual improvement of the ecological water supply system in the main urban area of Guangzhou, the increasing awareness of water resource conservation and protection and the strong water resource management system. To sum up, these characteristics were in good agreement with the above phenomenon (Fig. 5).

#### Spatial change of LULC based on grids

**Figure 7:**
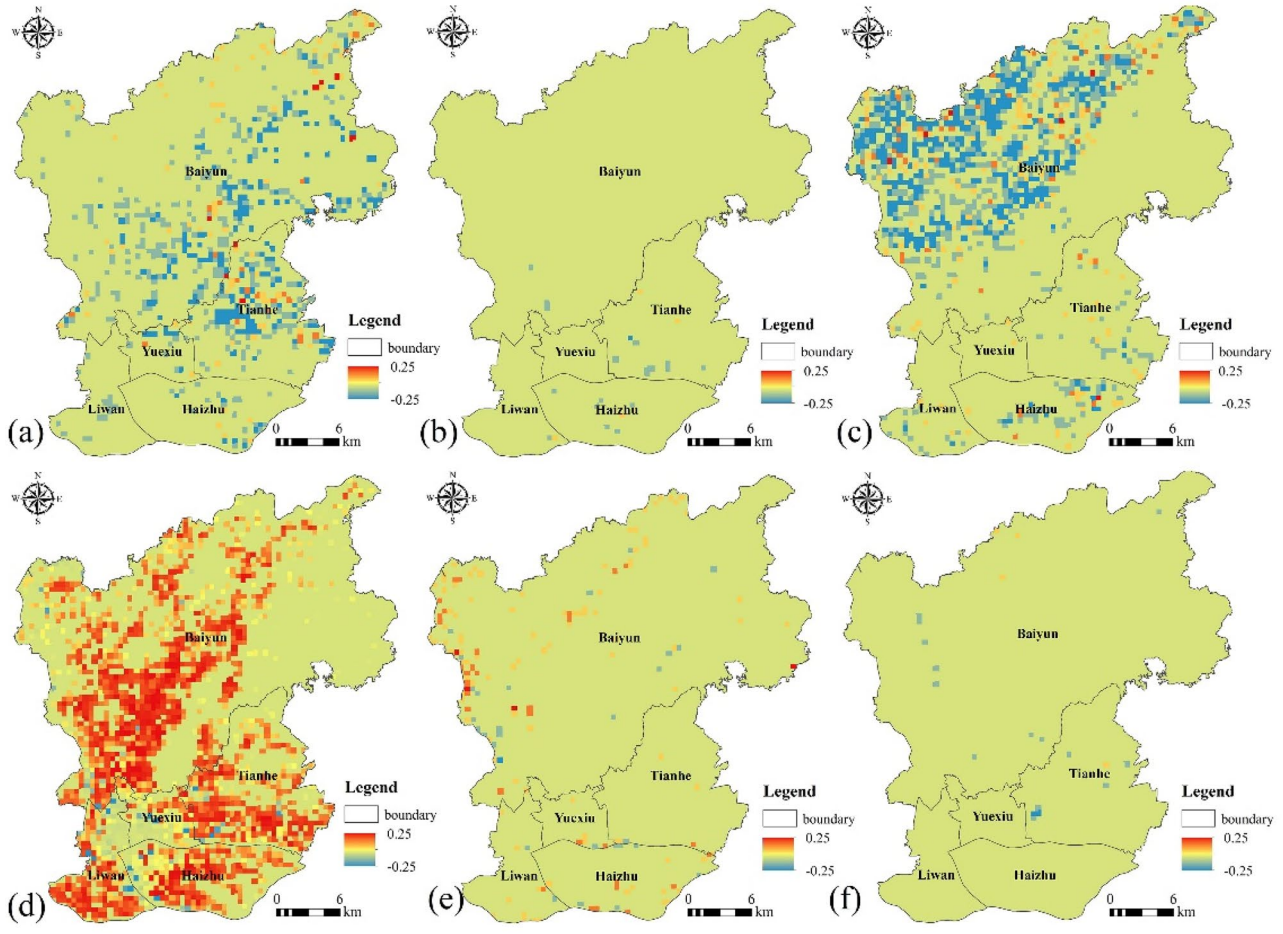
Spatial change of land cover types in the study area between 1987 and 2017. (**a**) forest, (**b**) grassland, (**c**) cultivated land, (**d**) built-up area (**e**), water body and (**f**) bare land. (Software: Arc Map 10.5.0, http://www.esri.com).

To clearly show the spatial changes of different LULC types from 1987 to 2017, the ArcGIS spatial statistics tool was used to calculate the grid-based change intensity. Here, the change map of each LULC type was subdivided into non-overlapping blocks of 0.5 km × 0.5 km, and the statistical information of land use change information in each grid was calculated (Fig. 7). The forest area in the Tianhe district and the Baiyun district decreased significantly (Fig. 7a), while the grassland change was not remarkable (Fig. 7b) and the cultivated land area in the northwest part of the Baiyun district showed a significant decrease (Fig. 7c). According to the change intensity chart, the main process of LULC change was the conversion between cultivated land, forest area and built-up area. Hotspots with shrinking forest and cultivated land were seriously threatened by the expansion of built-up areas (Fig. 7d). Water body and bare land areas did not change significantly (Fig. 7c,d). During the study period, the serious decrease in natural vegetation coupled with the decrease in the cultivated land ecosystem highlighted the serious decrease in important ecological categories of LUCC and the corresponding increase in production-oriented land use in the main urban area of Guangzhou. This means that human encroachment into the natural ecosystem increased.

### ESV change pattern

#### Change of total ESV

**Figure 8:**
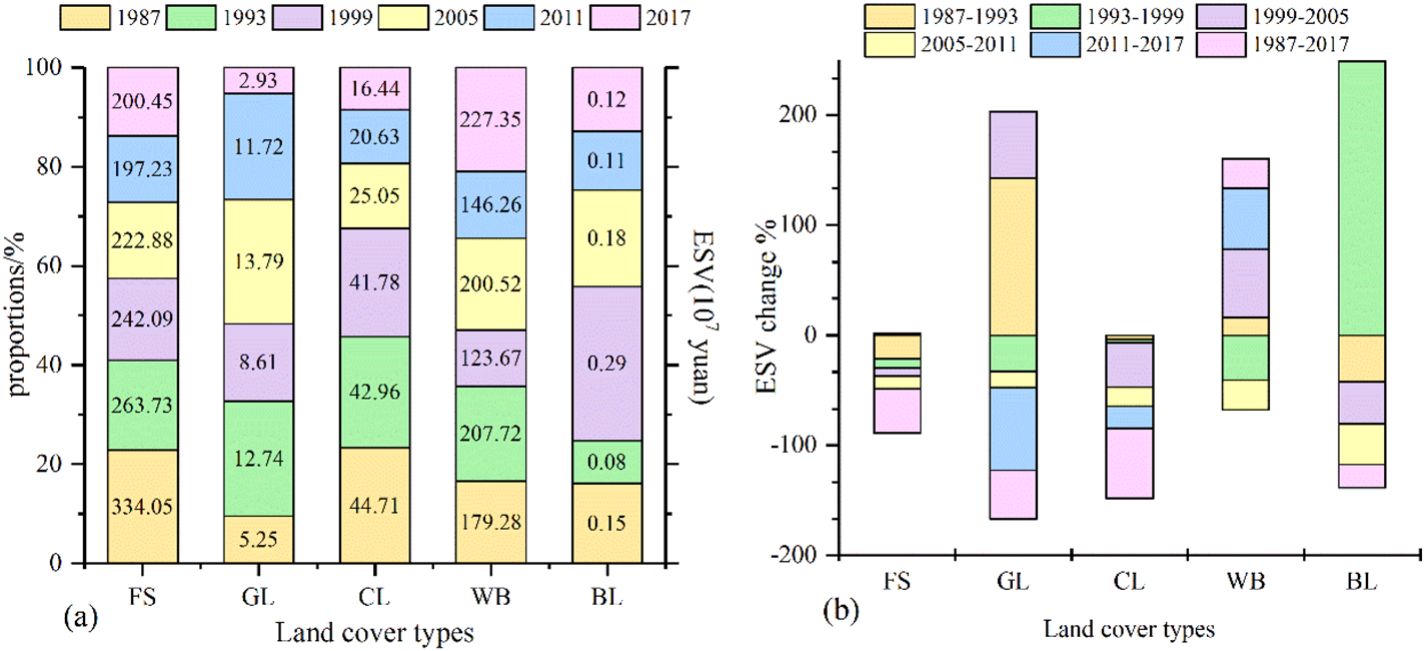
Changes in the total ESV for different land cover types in different periods.

In 1987, the total ESV in the main urban area of Guangzhou was estimated to be 5.63 × 10^9^ yuan (Fig. 8), and the ESV contribution of built-up areas was very weak. Therefore, an analysis was not carried out in this paper. The contribution of forest areas was the most important thing (about 59.29%), followed by water body areas (31.82%). The contribution of cultivated land was also larger (24.93%). The contribution of grassland ESV accounted for 0.93% of the total ESV, while the contribution of bare land to the total ESV was the smallest at only 0.03%.

The ESV decreased from 5.63 × 10^9^ yuan to 4.47 × 10^9^ yuan (Fig. 8) between 1987 and 2017. The total loss of ESV was mainly brought about by forest and cultivated land. The change in forest ESV accounted for 115.02%, followed by cultivated land, which accounted for 24.34%. In contrast, the total ESV of water body areas increased by 26.81% from 1.79 × 10^9^ yuan to 2.27 × 10^9^ yuan. In general, there was heterogeneity in ESV changes in different periods from 1987 to 2017 (Fig. 8). During the four periods of 1987–1993, 1993–1999, 1999–2005 and 2005–2011, the ESV of forest areas showed a decreasing trend. The largest percentage was 21.05% between 1987 and 1993, while from 2011 to 2017 the ESV of forest showed an increasing trend. The ESV of grassland showed the tend of “increase–decrease–increase–decrease–decrease”. The ESV of cultivated land continued to show a decreasing trend, while water body areas showed the trend of “increase–decrease–increase–decrease–increase”. The ESV of bare land fluctuated and increased significantly from 1993 to 1999. For the main urban area of Guangzhou, although water body areas were small, they contributed a lot to the ecosystem service function, the key factor in improving the regional ecological environment and the ecosystem service function.

#### Spatial change in ESV based on grids

**Figure 9:**
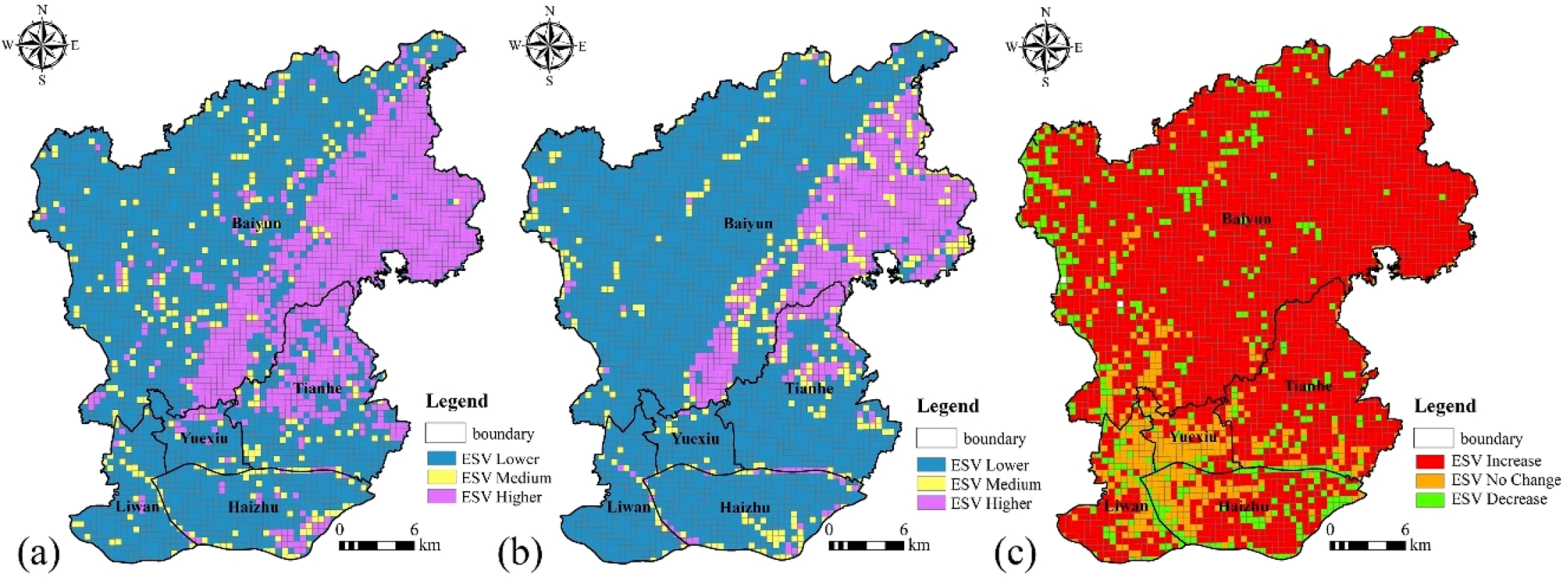
Changes in ESV based on a 0.5 km × 0.5 km grid between 1987 and 2017. (Software: Arc Map 10.5.0, http://www.esri.com).

By analysing the spatial change in ESV based on grids (Fig. 9), it can be seen that from 1987 to 2017 ESV changed to varying degrees. Between 1987 and 2017, the areas with higher ESVs decreased gradually, the areas with lower ESVs increased gradually and the areas with medium ESVs decreased gradually. The areas with higher ESVs were mainly distributed in the hilly area of the Baiyun district (mainly forest), the areas with lower ESVs were mainly distributed in the flat area of the main urban area and the areas with medium ESVs, including cultivated land, bare land and other ecosystem types (Fig. 9a,b). Based on Fig. 8c, from 1987 to 2017 the areas with decreased ESVs mainly consisted of areas with forest and grassland degradation. The total reduction areas of ESV were 787.25 km^2^, accounting for 74.46% from 1987 to 2017. Areas with increased ESV were concentrated and mainly distributed in the areas where bare land was converted into cultivated land or grassland, with an increase in area of 102.25 km^2^, accounting for 9.67%. The areas with unchanged ESVs were mainly found in the built-up areas of the Yuexiu, Haizhu and Liwan districts, with a total area of 167.75 km^2^, accounting for 15.87%.

#### Changes in the value of ecosystem service functions

We quantified the contribution of the ESV of each ecosystem function (Table 5). Based on the total amount of ESV from 1987 to 2017, the most important components were food production, climate regulation and water regulation. From 1987 to 2017, all ecosystem service functions (except water regulation) showed a downward trend. During the study period, the ESV of water supply declined faster than that of any other ecosystem service (− 179.33%); this was followed by food production (− 5.10%), nutrient cycling (− 1.86%), gas regulation (− 1.66%), raw materials (− 1.47%), climate regulation (− 4.30%) and soil conservation (− 1.33%). The ESV_f_ of aesthetic landscape had the lowest rate of change (− 0.59%). Of the reduced values, the reduction in water supply was the largest, followed by food production. In recent years, due to the large reduction in cultivated land area most cultivated land in the main urban area of Guangzhou consisted of paddy fields, resulting in a decline in the water supply value and food production value.

**Table 5:** Estimated values for different ecosystem functions (ESV_f)_ between 1987 and 2017.

Service project	1987	1993	1999	2005	2011	2017	Rate of interannual change	Level	Trend
Food production	52.737	1.942	1.806	1.291	1.067	0.974	− 5.10%	2	↓
Raw material	1.186	1.011	0.900	0.838	0.723	0.728	− 1.47%	5	↓
Water supply	− 1.044	− 0.858	− 1.342	0.098	− 0.014	0.722	− 179.33%	1	↓
Gas regulation	4.694	4.099	3.697	3.228	2.763	2.713	− 1.66%	4	↓
Climate regulation	10.817	9.093	8.077	7.691	6.668	6.737	− 1.42%	6	↓
Purifying environment	3.970	3.579	2.935	3.139	2.604	2.955	− 0.79%	9	↓
Water regulation	22.556	23.163	16.580	20.714	16.003	21.414	0.33%	11	↑
Soil conservation	4.400	3.725	3.270	3.194	2.758	2.822	− 1.33%	7	↓
Nutrient cycling	0.528	0.470	0.430	0.351	0.299	0.285	− 1.86%	3	↓
Biodiversity	4.850	4.352	3.582	3.801	3.167	3.558	− 0.85%	8	↓
Aesthetic landscape	2.330	2.145	1.710	1.898	1.557	1.822	− 0.59%	10	↓

From the perspective of ecosystem service functions, water supply, food production, nutrient cycling and gas regulation rank at the top. These four major ecological functions affected the total ESV the most in the main urban area of Guangzhou. The contribution rate of raw materials, food production and aesthetic landscape were quite low at only 13.06%. For this reason, this study considered the production function of the study area more important than the service function. It showed that the supply function (food production, raw materials and water supply) was the main driving force of ESV changes in the main urban area of Guangzhou.

#### Spatial change patterns in the value of ecosystem service functions

**Figure 10:**
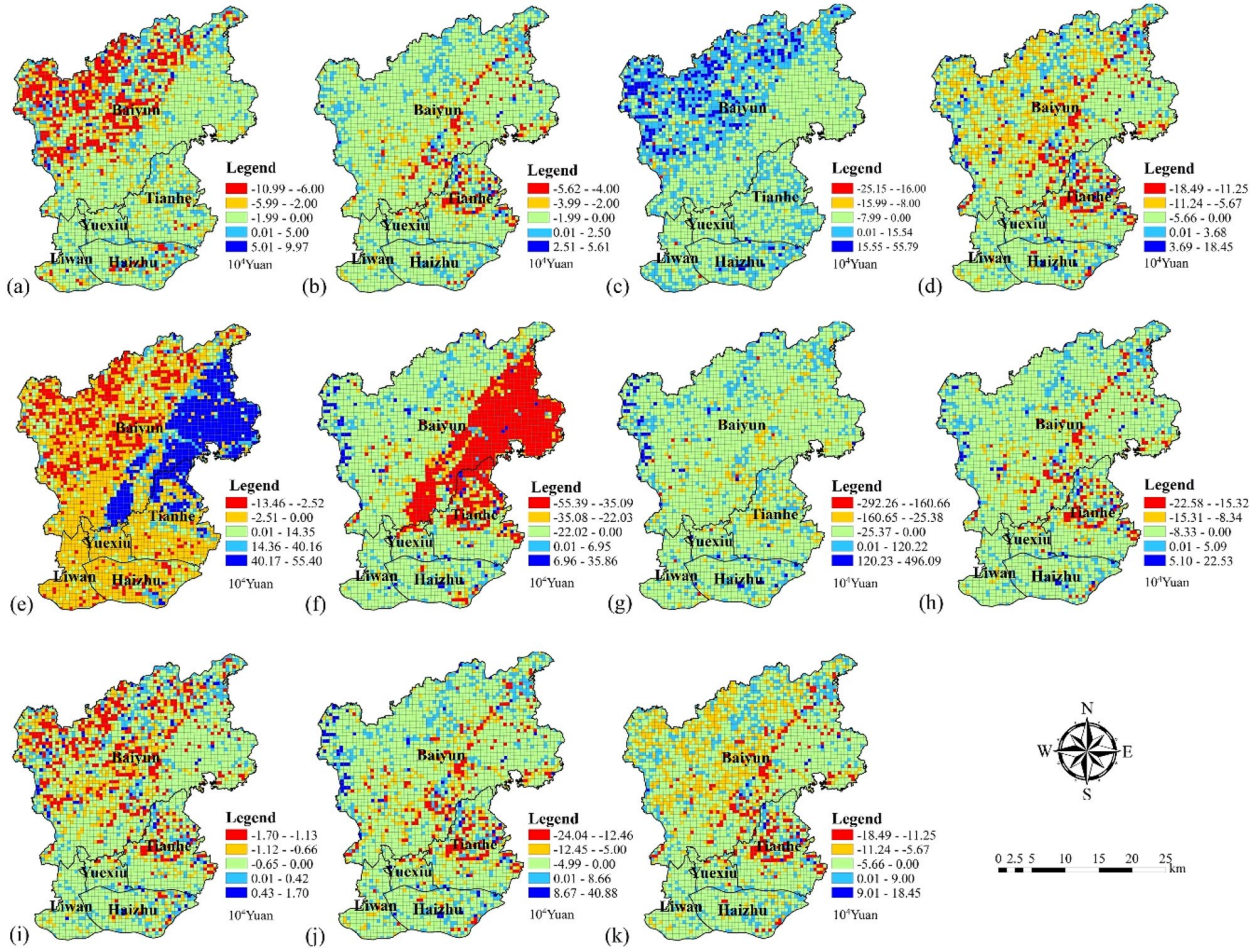
Value change for ecosystem service functions of (**a**) food production, (**b**) raw materials, (**c**) water supply, (**d**) gas regulation, (**e**) climate regulation, (**f**) purifying the environment, (**g**) water regulation, (**h**) soil conservation, (**i**) nutrient cycling, (**j**) biodiversity and (**k**) aesthetic landscape. (Software: Arc Map 10.5.0, http://www.esri.com).

The rate of change in the value of ecosystem service functions (ESV_f_) was different in different areas (Fig. 10). The ESV_f_ of food production, gas regulation, climate regulation, nutrient cycling and aesthetic landscape declined significantly in the northwest part of the Baiyun district. On the contrary, the ESV_f_ of water supply increased considerably in the study area. The ESV_f_ for climate regulation increased significantly in the northeast part of the Baiyun district, while the ESV_f_ for purifying the environment decreased significantly. In the north part of the Tianhe district, the ESV_f_ of gas regulation, purifying the environment, soil conservation, nutrient cycle maintenance, biodiversity and aesthetic landscape was significantly reduced.

#### Sensitivity analysis of ESV to value coefficient

As can be seen in Table 6, from 1987 to 2017 the sensitivity coefficient of the value coefficient of each land use type ranged from 0 to 0.6, all being less than 1. The sensitivity coefficient of forest areas was the highest at 0.59. That is, when the ESV of forest areas increased or decreased by 1%, the total ESV increased or decreased by 59%. Setting the value coefficient of built-up land and bare land did not affect ESV, and the sensitivity coefficient was 0. Accordingly, the sensitivity coefficient of grassland, cultivated land and water body areas increased from 0.02 to 0.51. The analysis showed that forest, water body areas and cultivated land played a vital role in ecosystem services. Regarding the accuracy of ESV from 1987 to 2017, the value coefficient was significant, indicating that the ESV index used in the study area was good, the ESV aggregate index was inelastic and the study results were reliable.

**Table 6:** Sensitivity index of ESV coefficients.

Change of value coefficients	1987		1993		1999		2005		2011		2017	
	%	CS	%	CS	%	CS	%	CS	%	CS	%	CS
Forest 50%	± 11.79	± 0.5929	± 11.36	± 0.5002	± 11.73	± 0.5813	± 11.28	± 0.4820	± 11.48	± 0.5246	± 11.13	± 0.4482
Grassland ± 50%	± 9.13	± 0.0093	± 9.20	± 0.0242	± 9.18	± 0.0207	± 9.23	± 0.0298	± 9.23	± 0.0312	± 9.12	± 0.0066
Cultivated land ± 50%	± 9.45	± 0.0794	± 9.46	± 0.0815	± 9.55	± 0.1003	± 9.34	± 0.054	± 9.34	± 0.054	± 9.26	± 0.0368
Water body ± 50%	± 10.54	± 0.3182	± 10.8	± 0.394	± 10.4	± 0.2970	± 11.6	± 0.4336	± 10.86	± 0.3890	± 11.40	± 0.5083
Bare land ± 50%	± 9.09	± 0.0003	± 9.09	± 0.0002	± 9.09	± 0.0007	± 9.09	± 0.0004	± 9.09	± 0.0003	± 9.09	± 0.0003

#### Driving force analysis of ecosystem service value

The ESV change was not only the result of land use transfer but also of other natural and socio-economic factors^33,34^. Therefore, it is necessary to analyse the influence of natural and socio-economic factors on the spatial differentiation of ESV change in the main urban area of Guangzhou.

Taking the ESV variable as the geo-detector dependent variable and ten natural and socio-economic indicators as independent variables, the single-factor contribution and factor interaction were quantitatively analysed using a geo-detector tool to explore the dominant factors of the spatial differentiation of ESV change in LULC types (Tables 7 and 8). The results of the single-factor driving force analysis showed that the ESV change for forest, water body and bare land areas was mainly restricted by natural and socio-economic factors (Table 7)^35^. The significance test of each factor of grassland and cultivated land is greater than 0.05, and these factors had no significant effect on the change in ESV. Single-factor analysis can reveal the dominant factors of the spatial differentiation of the ESV for LULC types in the main urban area of Guangzhou, but the spatial differentiation of the ESV was influenced by the interaction of multiple factors.

**Table 7:** Geographic detection results of ESV for each land use type.

ESV types	Driving forces	q	p	ESV types	Driving forces	q	p
Forest	NDVI	0.2147	0.0000	Cultivated land	NDVI	0.0038	0.0937
	Population	0.0547	0.0000		Population	0.0022	0.0625
	Elevation	0.6923	0.0000		Elevation	0.0004	0.9888
	Slope	0.3862	0.0000		Slope	0.0020	0.5138
	Distance from road	0.0473	0.0000		Distance from road	0.0018	0.5008
	Distance from railway	0.4258	0.0000		Distance from railway	0.0007	0.9270
	Land cover type	0.6167	0.0000		Land cover type	0.0036	0.0499
	GDP	0.0547	0.0000		GDP	0.0002	0.6250
	Secondary industry GDP	0.0519	0.0000		Secondary industry GDP	0.0005	0.5921
	Investment in fixed assets	0.0520	0.0000		Investment in fixed assets	0.0001	0.5921
Grassland	NDVI	0.0012	0.8421	Water body	NDVI	0.0306	0.0000
	Population	0.0076	0.0000		Population	0.1045	0.0000
	Elevation	0.0011	0.8221		Elevation	0.1393	0.0000
	Slope	0.0036	0.1647		Slope	0.1497	0.0000
	Distance from road	0.0006	0.9654		Distance from road	0.0126	0.0000
	Distance from railway	0.0053	0.0077		Distance from railway	0.0671	0.0000
	Land cover type	0.0020	0.3252		Land cover type	0.1211	0.0000
	GDP	0.0077	0.0000		GDP	0.1045	0.0000
	Secondary industry GDP	0.0015	0.1143		Secondary industry GDP	0.0462	0.0000
	Investment in fixed assets	0.0015	0.1143		Investment in fixed assets	0.0462	0.0000
Bare land	NDVI	0.3905	0.0000				
	Population	0.0356	0.0000				
	Elevation	0.0333	0.0000				
	Slope	0.0270	0.0000				
	Distance from road	0.0108	0.0000				
	Distance from railway	0.0053	0.0071				
	Land cover type	0.3398	0.0000				
	GDP	0.0356	0.0000				
	Secondary industry GDP	0.0356	0.0000				
	Investment in fixed assets	0.0356	0.0000

**Table 8:** Influence of interaction of spatial driving forces on ESV for each land use type.

ESV types	qmax(C = A + B)	Relationship	Interaction
Forest	Elevation ∩ Land cover type = 0.7935	C < A + B	Two-factor enhancement
Grassland	Distance from railway ∩ Slope = 0.0489	C > A + B	Nonlinear enhancement
Cultivated land	Distance from road ∩ Population = 0.0333	C > A + B	Nonlinear enhancement
Water body	GDP ∩ Elevation = 0.3222	C > A + B	Nonlinear enhancement
Bare land	Land cover type ∩ NDVI = 0.4959	C < A + B	Two-factor enhancement

A geo-detector (“interaction detector”) was used to analyse the interaction of driving factors in the spatial distribution of ESV (Table 8)^34^. The results of interactive detection showed that the interaction of any two factors was greater than any single factor, indicating that the ESV change in the main urban area of Guangzhou was the result of the comprehensive action of multiple factors. The interaction between elevation and land use types made the greatest contribution to the change in forest ESV, and the interaction of distance from railway and slope had the greatest influence on the change in grassland ESV. The interaction between distance from road and population was the biggest reason for the change in cultivated land ESV, and the interaction between GDP and elevation was the most important factor in the change in water body ESV.

## Discussion

### The ESV in response to LULC dynamics

Previous studies have shown that LUCC affects the main ecological processes of the ecosystem, such as energy exchange; the water cycle; soil erosion and accumulation; and the biogeochemical cycle, thus influencing ecosystem services^36–40^. Based on previous studies and this study, in the Zhujiang Delta we can see that the reduction in cultivated land and the expansion of built-up areas were the main characteristics of LULC changes^23,33^, rapid industrialization, urbanization and population growth were a major driver of LULC changes in the Guangzhou^24,37^. As one of the regions furthest advanced in implementing the Reform and Open Policy in China, the main urban area of Guangzhou experienced large-scale economic development, population growth and urban sprawl. In this study, based on the value equivalent factor per unit area improved by Xie^2^, we calculated the ESV of each land cover type from 1987 to 2017 and found that the total ESV decreased by 20.61% (1.162 billion yuan). These changes were primarily due to the reduction in ecosystem services caused by the reduction in forest area and cultivated land (Figs. 7 and 8)^41^. Although the water body areas were very small, they made a notable contribution to the ecosystem service function that was the key factor in improving the regional ecological environment and the ecosystem service function (Fig. 8)^40^. During the study period, most of the cultivated land and part of the forest areas were converted into built-up areas, resulting in a 63.23% decrease in the cultivated land ESV and a 39.99% decrease in forest area ESV (Fig. 7). This showed that the loss of forest area and cultivated land led directly to the decline in the ESVs in the main urban area of Guangzhou. This is consistent with the results of Ayanlade^42^, who found that the degradation of forests contributed the most to the reduction in ESV.

Our research revealed that the decline in ESV was mainly related to the huge decrease in forest area and cultivated land. Forests are considered to be the foremost providers of ecosystem services^43^. Studies in other regions of the world that reported a decrease in forest areas and that had an ESV assessment procedure similar to ours have reported a decrease in total ESV^6,14^. For example, Hu et al.^44^ pointed out that the decrease in forest area in southwest China led to a sharp decrease in ESV. Leh et al.^45^ also reported a decrease in ESVs between 2000 and 2009 as a result of altered forest ecosystems in West Africa. All these studies further confirm the finding of this study, which is that changes in LULC types have led to a significant loss of ESV.

The conversion of cultivated land to built-up areas has largely led to a reduction in food production and raw materials^15^ and a reduction in soil formation, biological control and water supply services. Among the 11 specific ecosystem services considered in this study, 90% of the services decreased with the decrease in revenue, and most of these services were supplied by natural ecosystems. This is in keeping with the findings of many studies around the world showing that urban expansion has negatively impacted the provision of other key ecosystem services, such as nutrient cycling; climate and water regulation^6^; food production and biological control^46^ and erosion control and water treatment^14^. In addition, urban expansion can also lead to environmental hazards^46^.

In order to more clearly explore the impact of LUCC on ESV value, this study applied correlation analysis to quantify the relationship between LUCC and ESV_f_. Land ecosystem services were classified into four types—provisioning services, regulating services, cultural services and supporting services (Table 9). The results revealed that there was a significant positive correlation between forest and cultivated land and ESV, while there was a positive correlation between water body areas and ESV (except supply services). Grassland and bare land were negatively correlated with ESV, mainly due to the small area of grassland and bare land in the main urban area of Guangzhou. Therefore, the changes in forest, cultivated land and water body areas had the greatest influence on the ESV. The classical sensitivity analysis also showed that LULC types such as forest, water body and cultivated land, played an important part in ecosystem services (Table 6). For the study of various land use types, the estimated ESV was inelastic relative to the value coefficient. Despite the fact that the value coefficient was uncertain, our estimation was relatively robust. Therefore, our assessment was applicable for calculating the value of long-term ecosystem services.

**Table 9:** The impact of LUCC on the ecosystem service value (1987–2017).

Types	Forest	Grassland	Cultivated land	Water body	Bare land
Provisioning service	0.8675	− 0.4437	0.4875	− 0.0016	− 0.0785
Regulating service	0.8365	− 0.1692	0.6233	0.4992	− 0.3084
Supporting service	0.9759	− 0.1343	0.8102	0.1527	− 0.0925
Cultural service	0.8741	− 0.1790	0.6359	0.4469	− 0.2897
Total value	0.9216	− 0.3978	0.5636	0.1121	− 0.1328

### Limitations of the study

In this study, the co-evolution of LULC and ESV was quantified, and the impact of land use evolution on the change in ESV was analysed in detail in the main urban area of Guangzhou. The conclusion was helpful to provide a scientific basis for the efficient allocation of land resources; rational regional planning and sustainable development; the scientific management of ecosystems; and the scientific formulation of ecological compensation policies in Guangzhou. However, there are several limitations of the methodology of benefit transfers which we have adopted here. For example, a major error is that by generalizing the unit values derived from 2011 year for a specific good as average unit values in all different years, the approach assumes homogeneity of ecosystems services value within the entire biome/LULC types in different years^13,14,45,47^. Due to the limitations of remote sensing images, LULC was not classified in enough detail. For example, in the data set, there was no difference between dry land and paddy land. Therefore, we used cultivated land as a representative and estimated the ESV of cultivated land rather than using separate calculations based on dry land and paddy land^14,48^. In addition, the ESV in built-up areas was not considered in this study because there was no suitable equivalent factor. In this paper, the coefficient of evaluating ESV was based on the equivalent value of the Chinese terrestrial ecosystem service proposed by Xie^2^, and combined with the characteristics of the research area, it was adjusted. However, the accuracy of estimating ESV in the research area still needs to be strengthened in the future.

## Conclusion

This study serves as a much-needed first assessment of the impact of LULC dynamics on ESV in the main urban area of Guangzhou. Our results showed that LULC significantly changed; there was significant expansion of built-up areas and a decrease in forest and cultivated land areas between 1987 and 2017. The proportion of forest and cultivated land decreased from 43.12% and 34.23% to 25.88% and 12.59%, respectively. Hotspots with shrinking forest and cultivated land were seriously threatened by the expansion of built-up areas. All LULC types were frequently converted in the study area. The total ESV decreased by 1.16 billion yuan (20.61%) from 1987 to 2017, the total loss of ESV was mainly brought about by forest and cultivated land. Water body contributed a lot to the ecosystem service function, the key factor in improving the regional ecological environment and the ecosystem service function. The effects of LULC changes on the specific ecosystem services are equally significant. Water supply, food production, nutrient cycle maintenance and gas regulation were the four major ecological functions that affected the total ESV in the main urban area of Guangzhou. Forest, water body and cultivated land types played a crucial role in ecosystem services. The ESV change was also restricted by natural factors and socio-economic factors, the interaction between elevation and land use types made the greatest contribution to the change in forest ESV, and the interaction between distance from road and population was the biggest reason for the change in cultivated land ESV, and the interaction between GDP and elevation was the most important factor in the change in water body ESV. During the study period, the serious decrease in natural vegetation coupled with the decrease in cultivated land highlighted the serious decrease in land use in the main urban area of Guangzhou, the important ecological categories of land use change and the corresponding increase in production-oriented land use. All this signifies that human encroachment on the natural ecosystem increased. Therefore, great attention should be paid to the protection of forest, cultivated land and water body. Moreover, proper land development and the maximum use of existing land resources can slow down the great changes in land and maintain the local ecological structure. At the same time, we should make full use of water resources, prevent soil erosion, stabilize food production, maintain a healthy nutrition cycle, reduce polluting gas emissions, ensure normal gas supply, and issue relevant regulations to slow down artificial intervention in the natural ecological environment, so as to alleviate the reduction of local ESV and promote the green development of Guangzhou. Last but not least, the government should facilitate payment for ecosystem services as a conservation strategy. The study can also help identify where and what LUCC are required to meet ecosystem services sustainability targets and minimize future risk to ESV.

## References

[1] Daily GC (1997). Nature’s Services: Societal Dependence on Natural Ecosystems.

[2] Xie GD, Zhang CX, Zhang CS, Xiao Y, Lu CX (2015). The value of ecosystem services in China. Resour. Sci..

[3] Costanza R, d'Arge R, de Groot R, Farber S, Grasso M, Hannon B, Limburg K, Naeem S, O'Neill RV, Paruelo J, Raskin RG, Sutton P, van den Belt M (1997). The value of the world's ecosystem services and natural capital. Nature.

[4] Xie GD, Zhang YL, Lu CX, Zheng D, Cheng SK (2001). Study on valuation of rangeland ecosystem services of China. J. Nat. Resour..

[5] Ellis E, Pontius R, Cleveland CJ (2007). Land-use and land-cover change. Encyclopedia of Earth. Environmental Information Coalition.

[6] Arowolo AO, Deng XZ, Olatunji OA, Obayelu AE (2018). Assessing changes in the value of ecosystem services in response to land-use/land-cover dynamics in Nigeria. Sci. Total Environ..

[7] Costanza R, Groot RD, Sutton P, Ploeg SVD, Anderson SJ, Kubiszewski L, Farber S, Turner RK (2014). Changes in the global value of ecosystem services. Glob. Environ. Change..

[8] Bagstad KJ, Reed JM, Semmens DJ, Sherrouse BC, Troy A (2016). Linking biophysical models and public preferences for ecosystem service assessments: A case study for the southern rocky mountain. Reg. Environ. Change.

[9] Yueriguli K, Yang ST, Zibibula S (2019). Impact of land use change on ecosystem service value in Ebinur Lake Basin, Xinjiang. Trans. Chin. Soc. Agric. Eng..

[10] Turner BL, Janetos AC, Verburg PH, Murray AT (2013). Land system architecture: Using land systems to adapt and mitigate global environmental change. Glob. Environ. Change..

[11] Hecht R, Behnisch M, Herold H (2020). Innovative approaches, tools and visualization techniques for analysing land use structures and dynamics of cities and regions (editorial). J. Geovis. Spat. Anal..

[12] Newman G, Hollander JB, Lee J (2018). Smarter shrinkage: A neighborhood-scaled rightsizing strategy based on land use dynamics. J. Geovis. Spat. Anal..

[13] Su WZ, Gu CL, Yang GS, Chen S, Zhen F (2010). Measuring the impact of urban sprawl on natural landscape pattern of the Western Taihu Lake watershed, China. Landsc. Urban Plann..

[14] Kindu M, Schneider T, Teketay D, Knoke T (2016). Changes of ecosystem service values in response to land use/land cover dynamics in Munessa-Shashemene landscape of the Ethiopian highlands. Sci. Total Environ..

[15] Lawler JJ, Lewis DJ, Nelson E, Plantinga AJ, Polasky S, Withey JC, Helmers DP, Martinuzzi S, Pennington D, Radeloff VC (2014). Projected land-use change impacts on ecosystem services in the United States. Proc. Natl. Acad. Sci. U.S.A..

[16] Yi H, Güneralp B, Filippi AM, Kreuter UP, Güneralp İ (2017). Impacts of land change on ecosystem services in the San Antonio River basin, Texas, from 1984 to 2010. Ecol. Econ..

[17] Hu CX, Guo XD, Lian G, Zhang ZM (2017). Effect of land use change on ecosystem service value in rapid urbanization areas of Yangtze river delta—a case study of Jiaxing city. Resourc. Environ. Yangtze Basin..

[18] National Bureau of Statistics (2018). China Statistical Yearbook.

[19] Liu XW, Zhang DX, Chen BM (2008). Characteristics of China’s town-level land use in rapid urbanization stage. Acta Geograph. Sin..

[20] Yang WR, Li F, Wang RS, Xiong XX, Liu AS (2013). Eco-service efficiency assessment method of urban land use: A case study of Changzhou City China. Acta Ecol. Sin..

[21] Chen W, Yan DS, Sun W (2015). Analyzing the patterns and processes of new urbanization development in the Yangtze River Delta. Geogr. Res..

[22] Li Y, Cui HS (2011). Spatio-temporal analysis of main urban Land-use patterns in Guangzhou. Territory Nat. Resour. Study..

[23] He Y, Dou P, Yan HW, Zhang LF, Yang SW (2018). Quantifying the main urban area expansion of Guangzhou using Landsat imagery. Int. J. Remote Sens..

[24] Ye YQ, Bryan BA, Zhang JE, Connor JD, Chen LL, Qin Z, He MQ (2018). Changes in land-use and ecosystem services in the Guangzhou-Foshan Metropolitan Area, China from 1990 to 2010: Implications for sustainability under rapid urbanization. Ecol. Ind..

[25] Gao PP, Li YP, Gong JW, Huang GH (2021). Urban land-use planning under multi-uncertainty and multiobjective considering ecosystem service value and economic benefit—A case study of Guangzhou China. Ecol. Complex..

[26] Hu MM, Li ZT, Wang YF, Jiao MY, Li M, Xia BC (2019). Spatio-temporal changes in ecosystem service value in response to land-use/cover changes in the Pearl River Delta. Resour. Conserv. Recycl..

[27] Ye YQ, Zhang JE, Chen LL, Yang YO, Prem P (2015). Dynamics of ecosystem service values in response to landscape pattern changes from 1995 to 2005 in Guangzhou, southern china. Appl. Ecol. Environ..

[28] Guangzhou Statistics Bureau (2019). Guangzhou Statistical Yearbook 2019.

[29] Xu H (2008). A new index for delineating built-up land features in satellite imagery. Int. J. Remote Sens..

[30] Xu H (2006). Modification of normalised difference water index (NDWI) to enhance open water features in remotely sensed imagery. Int. J. Remote Sens..

[31] Dou P, Chen Y (2017). remote sensing imagery classification using AdaBoost with a weight vector (WVAdaBoost). Remote Sens. Lett..

[32] Wang JF (2017). Geo-detector: Principle and prospective. Acta Geogr. Sin..

[33] Liu YL, Cheng FL, Li F, Yang MZ, Zhao GW (2019). Interactive detection between evolutional features and driving forces of guangzhou’s landscape pattern. Resour. Ind..

[34] Huang MY, Yue WZ, Fang B, Feng SR (2019). Scale response characteristics and geographic exploration mechanism of spatial differentiation of ecosystem service values in Dabie Mountain area, central China from 1970 to 2015. Acta Geogr. Sin..

[35] Liu XP, Chen X, Hua KP, Wang YJ, Wang P, Han XJ, Ye JY, Wen SQ (2018). Effects of land use change on ecosystem services in arid area ecological migration. Chin. Geogr. Sci..

[36] Fu B, Wang S, Su C, Forsius M (2013). Linking ecosystem processes and ecosystem services. Curr. Opin. Environ. Sustain..

[37] Liu HX, Li YP, Yu L (2019). Urban agglomeration (Guangzhou-Foshan-Zhaoqing) ecosystem management under uncertainty: A factorial fuzzy chance-constrained programming method. Environ. Res..

[38] Fu BJ, Zhang LW (2014). Land use change and ecosystem services: Concepts, methods and progress. Adv. Geogr. Sci..

[39] Zhang F, Yushanjiang A, Jing Y (2019). Assessing and predicting changes of the ecosystem service values based on land use/cover change in Ebinur Lake Wetland National Nature Reserve, Xinjiang, China. Sci. Total Environ..

[40] Xu ZH, Fan WG, Wei HJ, Zhang P, Ren JH, Gao ZC, Ulgiati S, Kong WD, Dong XB (2019). Evaluation and simulation of the impact of land use change on ecosystem services based on a carbon flow model: A case study of the Manas River Basin of Xinjiang China. Sci. Total Environ..

[41] Fan XC, Liu JB, Chen JZ, Zhao LL (2018). Changes of land use and functions of ecosystem service: A case study in China. Pol. J. Environ. Stud..

[42] Ayanlade A (2012). Evaluating environmental change impacts on ecological services in the Niger Delta of Nigeria. Ife Res. Publ. Geogr..

[43] Martínez ML, Perez-Maqueo O, Vazquez G, Castillo-Campos G, García-Franco J, Mehltreter K, Equihua M, Landgrave R (2009). Effects of land use change on biodiversity and ecosystem services in tropical montane cloud forests of Mexico. For. Ecol. Manag..

[44] Hu H, Liu W, Cao M (2008). Impact of land use and land cover changes on ecosystem services in Menglun, Xishuangbanna, Southwest China. Environ. Monit. Assess..

[45] Leh MD, Matlock MD, Cummings EC, Nalley LL (2013). Quantifying and mapping multiple ecosystem services change in West Africa. Agric. Ecosyst. Environ..

[46] Ubuoh EA, Uka A, Egbe C (2016). Effects of flooding on soil quality in Abakaliki agroeco-logical zone of South-Eastern State, Nigeria. Int. J. Environ. Chem. Ecotoxicol. Res..

[47] Schmidt S, Manceur AM, Seppelt R (2016). Uncertainty of monetary valued ecosystem services–value transfer functions for global mapping. PLoS ONE.

[48] Gashaw T, Tulu T, Argaw M, Worqlul AW, Tolessa T, Kindu M (2018). Estimating the impacts of land use/land cover changes on ecosystem service values: The case of the Andassa watershed in the Upper Blue Nile basin of Ethiopia. Ecosyst. Serv..

